# Clinical significance of *CDKN2A* homozygous deletion in combination with methylated *MGMT* status for *IDH*‐wildtype glioblastoma

**DOI:** 10.1002/cam4.3860

**Published:** 2021-04-10

**Authors:** Yusuke Funakoshi, Nobuhiro Hata, Kosuke Takigawa, Hideyuki Arita, Daisuke Kuga, Ryusuke Hatae, Yuhei Sangatsuda, Yutaka Fujioka, Aki Sako, Toru Umehara, Tadamasa Yoshitake, Osamu Togao, Akio Hiwatashi, Koji Yoshimoto, Toru Iwaki, Masahiro Mizoguchi

**Affiliations:** ^1^ Department of Neurosurgery Graduate School of Medical Sciences Kyushu University Fukuoka Japan; ^2^ Department of Neurosurgery Graduate School of Medicine Osaka University Suita Japan; ^3^ Department of Clinical Radiology Graduate School of Medical Sciences Kyushu University Fukuoka Japan; ^4^ Department of Neurosurgery Graduate School of Medical and Dental Sciences Kagoshima University Kagoshima Japan; ^5^ Department of Neuropathology Graduate School of Medical Sciences Kyushu University Fukuoka Japan

**Keywords:** CDKN2A, glioblastoma, IDH‐wildtype, MGMT, survival

## Abstract

**Objective:**

Accumulating evidence from recent molecular diagnostic studies has indicated the prognostic significance of various genetic markers for patients with glioblastoma (GBM). To evaluate the impact of such genetic markers on prognosis, we retrospectively analyzed the outcomes of patients with *IDH*‐wildtype GBM in our institution. In addition, to assess the impact of bevacizumab (BEV) treatment, we compared overall survival (OS) between the pre‐ and post‐BEV eras.

**Methods:**

We analyzed the data of 100 adult patients (over 18 years old) with *IDH*‐wildtype GBM from our database between February 2006 and October 2018. Genetic markers, such as *MGMT* methylation status, *EGFR* amplification, *CDKN2A* homozygous deletion, and clinical factors were analyzed by evaluating the patients’ OS.

**Results:**

*CDKN2A* homozygous deletion showed no significant impact on OS in patients with methylated *MGMT* status (*p* = 0.5268), whereas among patients with unmethylated *MGMT* status, there was a significant difference in OS between patients with and without *CDKN2A* homozygous deletion (median OS: 14.7 and 16.9 months, respectively, *p* = 0.0129). This difference was more evident in the pre‐BEV era (median OS: 10.1 and 15.6 months, respectively, *p* = 0.0351) but has become nonsignificant in the post‐BEV era (median OS: 16.0 and 16.9 months, respectively, *p* = 0.1010) due to OS improvement in patients with *CDKN2A* homozygous deletion. However, these findings could not be validated in The Cancer Genome Atlas cohort.

**Conclusions:**

*MGMT* and *CDKN2A* status subdivided our cohort into three race‐specific groups with different prognoses. Our findings indicate that BEV approval in Japan led to OS improvement exclusively for patients with concurrent unmethylated *MGMT* status and *CDKN2A* homozygous deletion.

## Graphical abstract

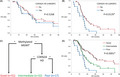

## 1 INTRODUCTION

To achieve accurate prognostic stratification, the genetic findings of glioblastoma (GBM) have been extensively studied, and numerous prognostic genetic markers reported. Recently, comprehensive molecular analyses of GBMs, such as The Cancer Genome Atlas (TCGA) projects, have revealed additional details regarding the molecular and genetic pathways in GBM tumorigenesis.^1^ Methylated *MGMT* status is a representative predictive factor in patients with GBM. MGMT protein removes alkyl groups from guanine at the O6 position, inhibiting the effect of alkylating drugs such as temozolomide (TMZ).^2^ MGMT promoter methylation transcriptionally silences gene expression, and leads to favorable outcomes in patients with GBM.^2^, ^3^, ^4^ Although methylated *MGMT* status is recognized as the most robust predictive marker for patients with GBM, other genetic markers associated with prognosis, including *EGFR* amplification, *TERT* promoter mutation, chromosome 10 loss, and *CDKN2A*/*B* homozygous deletion, have also been reported.^5^, ^6^, ^7^, ^8^, ^9^ The *CDKN2A*/*B* locus is found on chromosome 9p21. *CDKN2A* encodes proteins p14^ARF^ and p16^INK4a^, whereas *CDKN2B* encodes protein p15^INK4b^. These proteins function as tumor suppressors, and homozygous deletion of *CDKN2A*/*B* can contribute to uncontrolled tumor cell proliferation.^6^ *CDKN2A* homozygous deletion has been well‐analyzed in many tumors, including glioma, and frequently reported as a poor prognostic marker in patients with *IDH*‐mutant diffuse astrocytic glioma.^10^, ^11^, ^12^, ^13^, ^14^, ^15^, ^16^, ^17^ Additionally, in the Consortium to Inform Molecular and Practical Approaches to CNS Tumor Taxonomy—Not Official WHO (cIMPACT‐NOW) update 5, *IDH*‐mutant astrocytomas with *CDKN2A*/*B* homozygous deletion were classified into WHO grade 4 regardless of pathological findings.^18^ Other studies have also reported the *CDKN2A*/*B* homozygous deletion to be associated with unfavorable outcomes for all *IDH*‐mutant astrocytoma grades (WHO grades II–IV) and *IDH*‐wildtype GBM.^19^, ^20^, ^21^

To evaluate the impact of these genetic markers on the prognosis of patients with *IDH*‐wildtype GBM, we retrospectively analyzed the outcomes of *IDH*‐wildtype patients with GBM in our institution. We evaluated The Cancer Genome Atlas (TCGA) cohort for validation and showed the differences in molecular profile frequencies between these two cohorts, as well as the unique characteristics of GBM in Japanese patients. In addition, we previously reported that the optional first‐line bevacizumab (BEV) administration can prolong overall survival complementary to TMZ in a Japanese clinical setting.^22^ Therefore, the impact of BEV approval on prognosis in Japan based on genetic stratification was also evaluated.

## 2 METHODS

### 2.1 Patients

We analyzed the data of 100 adult patients (over 18 years old) with *IDH*‐wildtype GBM in our database between February 2006 and October 2018. Table 1 summarizes patients’ clinical characteristics. Patients who refused adjuvant treatment, had infratentorial tumors, or whose genetic status was unknown due to a lack of available tissue samples were excluded. Patients with *BRAF* and *H3F3A* mutations were also excluded, as they comprise a distinct biological GBM subgroup.^23^, ^24^, ^25^ All study participants provided informed consent. This study was approved by a local ethics committee, and conducted in accordance with the 1964 Declaration of Helsinki (as revised in Fortaleza, Brazil, October 2013).

**TABLE 1:** Patients’ clinical characteristics

Characteristics	All (*n* = 100)	Pre‐BEV (*n* = 45)	Post‐BEV (*n* = 55)	*p*‐value
Age (years)	65.0 (56.3–70.0)	63.0 (55.5–69.5)	66.0 (59.0–74.0)	0.1444
Gender				0.3149
Male	51 (51.0%)	20 (44.4%)	31 (56.4%)	
Female	49 (49.0%)	25 (55.6%)	24 (43.6%)	
KPS score (points)	80.0 (60.0–90.0)	70.0 (60.0–80.0)	90.0 (60.0–90.0)	0.0036*
Maximum tumor diameter (mm)	50.0 (37.0–60.8)	51.0 (45.0–64.0)	45.0 (28.0–60.0)	0.0111*
Resection				0.9670
GTR/STR	62 (62.0%)	28 (62.2%)	34 (61.8%)	
PR/Biopsy	38 (38.0%)	17 (37.8%)	21 (38.2%)	
BEV usage				<0.0001*
No	58 (58.0%)	39 (86.7%)	19 (34.5%)	
First‐line	20 (20.0%)	0 (0.0%)	20 (36.4%)	
Second‐line	22 (22.0%)	6 (13.3%)	16 (29.1%)	

Note

Data for age, KPS score, and maximum tumor diameter are presented as median (interquartile range).

Abbreviations: BEV, bevacizumab; KPS, Karnofsky Performance Status; GTR, gross total tumor removal; STR, subtotal tumor removal; PR, partial tumor removal.

* indicates statistical significance.

### 2.2 Treatment

Gross (GTR) or subtotal tumor removal (STR), defined as previously described,^22^ was performed in 62 (62.0%) patients. Partial tumor removal (PR) or biopsy was performed in 38 (38.0%) patients. Fluorescence‐guided surgery using 5‐aminolevulinic acid was used to determine the resection range in elective operations. Optional carmustine wafer implants were performed as previously described.^22^ After TMZ approval in 2006, GBM was treated using the Stupp regimen,^26^ and subsequent maintenance TMZ treatment was performed as described elsewhere.^22^ Since the approval of BEV, it has been applied in combination with the Stupp regimen for patients with severe clinical conditions such as unresectable tumors, low Karnofsky Performance Scale (KPS) scores, or advanced age; other patients were treated using the Stupp regimen and second‐line BEV administration after recurrence (post‐BEV era). Table 1 summarizes BEV usage in the two eras. Six (13.3%) patients in the pre‐BEV era underwent second‐line BEV because the recurrence occurred in the post‐BEV era. Although BEV treatment was generally performed according to the Avastin in Glioblastoma (AVAglio) regimen,^27^ BEV therapy was tapered or discontinued after approximately six months as per physician's decision based on the evaluation of improvements in clinical conditions and/or radiological findings. Concurrent radiotherapy was performed as previously described.^22^

### 2.3 Genetic analysis

Tissue samples and DNA were prepared as previously described,^22^ and genetic alterations identified as having prognostic potential in GBM were analyzed.^24^, ^25^, ^28^, ^29^, ^30^, ^31^ Hotspot mutations in the *IDH1*, *IDH2*, *BRAF*, *H3F3A* gene bodies, and *TERT* promoter were detected, and *MGMT* methylation status assessed as previously described.^32^, ^33^, ^34^ Copy number alterations (CNA), including those for genes *EGFR*, *CDKN2A*, *PTEN*, *PDGFR*, *CDK4*, *TP53*, were evaluated using a multiplex ligation‐dependent probe amplification (MLPA) kit (P105‐2; MRC‐Holland, Amsterdam, the Netherlands) containing *PDGFRA*, *EGFR*, *CDKN2A*, *PTEN*, *CDK4*, *MDM2*, *NFKBIA*, and *TP53* specific probes, with six other probes used as control probes (http://www.mlpa.com). MLPA was performed according to manufacturer's protocol. Denatured fragments were separated and quantified by electrophoresis using an ABI 3730 capillary sequencer (Applied Biosystems Nieuwerkerk aan de Ijssel, the Netherlands) and analyzed using GeneMapper^®^ (Applied Biosystem) and Coffalyser^®^ software (MRC‐Holland). Based on previous studies, we used thresholds of 1.2 and 0.8 for the detection of gains and losses, respectively.^35^, ^36^ In addition, ratios below 0.4, and above 2.0 were considered homozygous deletions, or amplifications, respectively.^35^

### 2.4 TCGA

To assess the accuracy of outcomes in our study, we performed a validation cohort study using TCGA database. We extracted the data of 577 patients with *IDH*‐wildtype GBM in TCGA from the publicly available cBioPortal for Cancer Genomics database (http://cbioportal.org) and the supplemental data of a previous publication by TCGA.^1^ The exclusion criteria were as similar as possible to our study. Infratentorial tumors seemed to be included, because no tumor location data were available. Of the 577 patients in the TCGA cohort, 144 patients receiving TMZ chemoradiation as initial treatment were selected. Patients initially treated with either radiation alone, TMZ alone, or an alkylating chemotherapy agent other than TMZ, along with patients for whom any such information was unavailable, were excluded, as were patients whose *MGMT* and *CDKN2A* status were unavailable. Information on BEV usage was not available. TCGA clinical information on GBM is publicly available, so approval of the local ethics committee was not necessary.

### 2.5 Statistical analysis

Statistical analyses were performed using the JMP software (version 14, SAS Institute). Clinical characteristics were evaluated using the chi‐square test, Fisher's exact test, and Mann–Whitney U‐test. Postoperative OS was evaluated using the Kaplan–Meier method. Differences in distributions were compared using the log‐rank test. Cox proportion hazards models were employed to estimate hazard ratios (HRs) and 95% confidence intervals (CIs) for the putative prognostic factors and genetic markers. Background differences between groups with and without *CDKN2A* homozygous deletion were analyzed using the chi‐square test or Fisher's exact test. To analyze the clinical impact of BEV approval in Japan, OS in the pre‐BEV and post‐BEV eras were compared. *p* < 0.05 was considered to indicate statistical significance.

## 3 RESULTS

### 3.1 Genetic and clinical prognostic factors

Table 2 shows the genetic markers analyzed as prognostic factors in this study. Unmethylated *MGMT* status was the only significant predictor of poor prognosis [HR: 2.29 (1.43–3.68), *p* < 0.0006 (univariate analysis); HR: 2.76 (1.66–4.60), *p* < 0.0001 (multivariate analysis)]. Although *CDKN2A* homozygous deletion was not significantly associated with poor prognosis in univariate analysis [HR: 1.40 (0.89–2.22), *p* = 0.1492], a significant difference was observed in multivariate analysis [HR: 1.73 (1.05–2.84), *p* = 0.0303]. Table 3 shows the clinical prognostic factors, including the significant genetic factors *MGMT* methylation and *CDKN2A* status. In univariate analysis, age, PR/biopsy, and unmethylated *MGMT* status were significantly associated with poor prognosis [HR: 1.86 (1.09–3.17), *p* = 0.0221; HR: 1.75 (1.10–2.79), *p* = 0.0181; and HR: 2.29 (1.43–3.68), *p* = 0.0006, respectively]. In multivariate analysis, age, unmethylated *MGMT* status, and *CDKN2A* homozygous deletion were significantly associated with poor prognosis [HR: 2.22 (1.25–3.94), *p* = 0.0065; HR: 2.86 (1.70–4.82), *p* < 0.0001; and HR: 1.76 (1.09–2.86), *p* = 0.0212, respectively]. In patients who underwent PR/biopsy, there was a trend of poor prognosis [HR: 1.52 (0.93–2.49), *p* = 0.0987]. There was no significant bias between groups with and without *CDKN2A* homozygous deletion (Table 4).

**TABLE 2:** Genetic prognostic factors

Genetic marker	Case (*n* = 100)	Univariate analysis		Multivariate analysis	
		HR (95% CI)	*p*‐value	HR (95% CI)	*p*‐value
Unmethylated *MGMT* status	49 (49.0%)	2.29 (1.43–3.68)	0.0006*	2.76 (1.66–4.60)	<0.0001*
*TERT* mutation	63 (63.0%)	0.96 (0.60–1.53)	0.8538	0.79 (0.45–1.36)	0.3888
*EGFR* amplification	19 (19.0%)	1.22 (0.69–2.17)	0.4871	1.35 (0.75–2.45)	0.3213
*CDKN2A* homozygous deletion	39 (39.0%)	1.40 (0.89–2.22)	0.1492	1.73 (1.05–2.84)	0.0303*
*PTEN* loss	58 (58.0%)	1.06 (0.66–1.68)	0.8174	0.93 (0.54–1.60)	0.7856
*PDGFRA* amplification	16 (16.0%)	1.14 (0.60–2.16)	0.6940	—	—
*CDK4* amplification	17 (17.0%)	0.76 (0.39–1.48)	0.4136	—	—
*TP53* loss	36 (36.0%)	1.09 (0.67–1.77)	0.7287	—	—

Abbreviations: CI, confidence intervalHR, hazard ratio.

* indicates statistical significance.

**TABLE 3:** Clinical and genetic prognostic factors

Prognostic Factor	Univariate analysis		Multivariate analysis	
	HR (95% CI)	*p*‐value	HR (95% CI)	*p*‐value
Age (>70 years)	1.86 (1.09–3.17)	0.0221*	2.22 (1.25–3.94)	0.0065*
KPS score (<80 points)	1.25 (0.79–1.98)	0.3336	1.10 (0.64–1.87)	0.7325
Maximum tumor diameter (>50 mm)	1.35 (0.85–2.12)	0.1998	1.38 (0.85–2.24)	0.1965
PR/biopsy	1.75 (1.10–2.79)	0.0181*	1.52 (0.93–2.49)	0.0987
Pre‐BEV era	0.88 (0.56–1.40)	0.5947	1.10 (0.67–1.82)	0.6975
Unmethylated *MGMT* status	2.29 (1.43–3.68)	0.0006*	2.86 (1.70–4.82)	<0.0001*
*CDKN2A* homozygous deletion	1.40 (0.89–2.22)	0.1492	1.76 (1.09–2.86)	0.0212*

Abbreviations: BEV, bevacizumab;CI, confidence interval; GTR, gross total tumor removal; HR, hazard ratio; KPS, Karnofsky Performance Status; PR, partial tumor removalSTR, subtotal tumor removal.

* indicates statistical significance.

**TABLE 4:** Background of patients with and without *CDKN2A* homozygous deletion

Prognostic factor	*CDKN2A* HD (‐) (*n* = 61)	*CDKN2A* HD (+) (*n* = 39)	*p*‐value
Age			1.000
<70 years	47 (77.1%)	30 (76.9%)	
>70 years	14 (22.9%)	9 (23.1%)	
KPS score			0.4182
>80 points	35 (57.4%)	19 (48.7%)	
<80 points	26 (42.6%)	20 (51.3%)	
Maximum tumor diameter			0.8398
<50 mm	33 (54.1%)	22 (56.4%)	
>50 mm	28 (45.9%)	17 (43.6%)	
Resection			0.4022
GTR/STR	40 (64.5%)	22 (56.4%)	
PR/biopsy	21 (35.5%)	17 (43.6%)	
Treatment era			0.5440
Pre‐BEV	29 (47.5%)	16 (41.0%)	
Post‐BEV	32 (52.5%)	23 (59.0%)	
*MGMT* status			0.4182
Methylated	29 (47.5%)	22 (56.4%)	
Unmethylated	32 (52.5%)	17 (43.6%)	

Abbreviations: *CDKN2A* HD, *CDKN2A* homozygous deletion; BEV, bevacizumab; KPS, Karnofsky Performance Status; GTR, gross total tumor removal; STR, subtotal tumor removal; PR, partial tumor removal.

### 3.2 Association of *CDKN2A* homozygous deletion with prognosis depending on *MGMT* status

**FIGURE 1:**
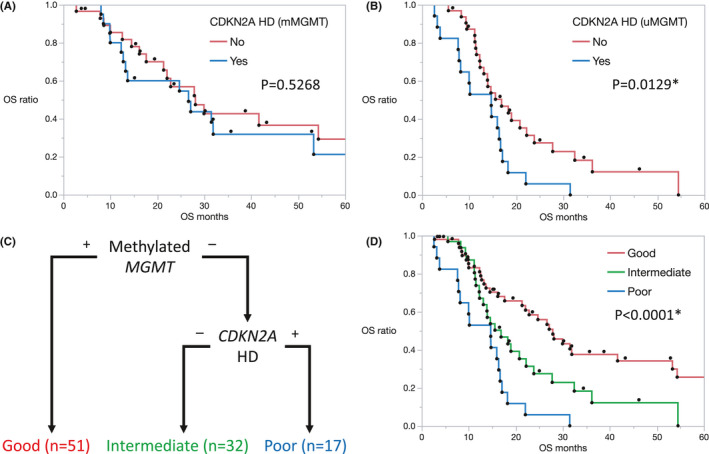
(A) Kaplan–Meier estimates of overall survival (OS) in patients of newly diagnosed glioblastoma (GBM) with methylated *MGMT* status in our cohort. Patients without and with *CDKN2A* homozygous deletion are represented by the red and blue lines, respectively. (B) Kaplan–Meier OS estimates in patients of newly diagnosed GBM with unmethylated *MGMT* status in our cohort. Patients without and with *CDKN2A* homozygous deletion are represented by the red and blue lines, respectively. (C) Flowchart shows revised grading system of *IDH*‐wildtype GBM. (D) Kaplan–Meier OS estimates in patients of newly diagnosed GBM in our cohort. Patients are classified into three groups with different prognosis. The good prognosis group includes patients with methylated *MGMT* status. The intermediate prognosis group includes patients with unmethylated *MGMT* status and without *CDKN2A* homozygous deletion. The poor prognosis group includes patients with unmethylated *MGMT* status and *CDKN2A* homozygous deletion. Patients in good, intermediate, and poor prognosis group are represented by the red, green, and blue lines, respectively. *indicates statistical significance. *CDKN2A* HD: *CDKN2A* homozygous deletion

In patients with methylated *MGMT* status, the median OS in patients with and without *CDKN2A* homozygous deletion was 26.6 and 28.1 months, respectively; however, the difference was not significant (*p* = 0.5268) (Figure 1A). In patients with unmethylated *MGMT* status, there was a significant difference in median OS between patients with and without *CDKN2A* homozygous deletion (14.7 and 16.9 months, respectively; *p* = 0.0129) (Figure 1B). Accordingly, patients with *IDH*‐wildtype GBM could be classified into three groups with different prognoses—good prognosis: patients with methylated *MGMT* status, intermediate prognosis: patients with unmethylated *MGMT* status and without *CDKN2A* homozygous deletion, and poor prognosis: patients with unmethylated *MGMT* status and *CDKN2A* homozygous deletion (Figure 1C). OS was significantly different among these three groups (*p* < 0.0001) (Figure 1D).

### 3.3 Validation cohort

**FIGURE 2:**
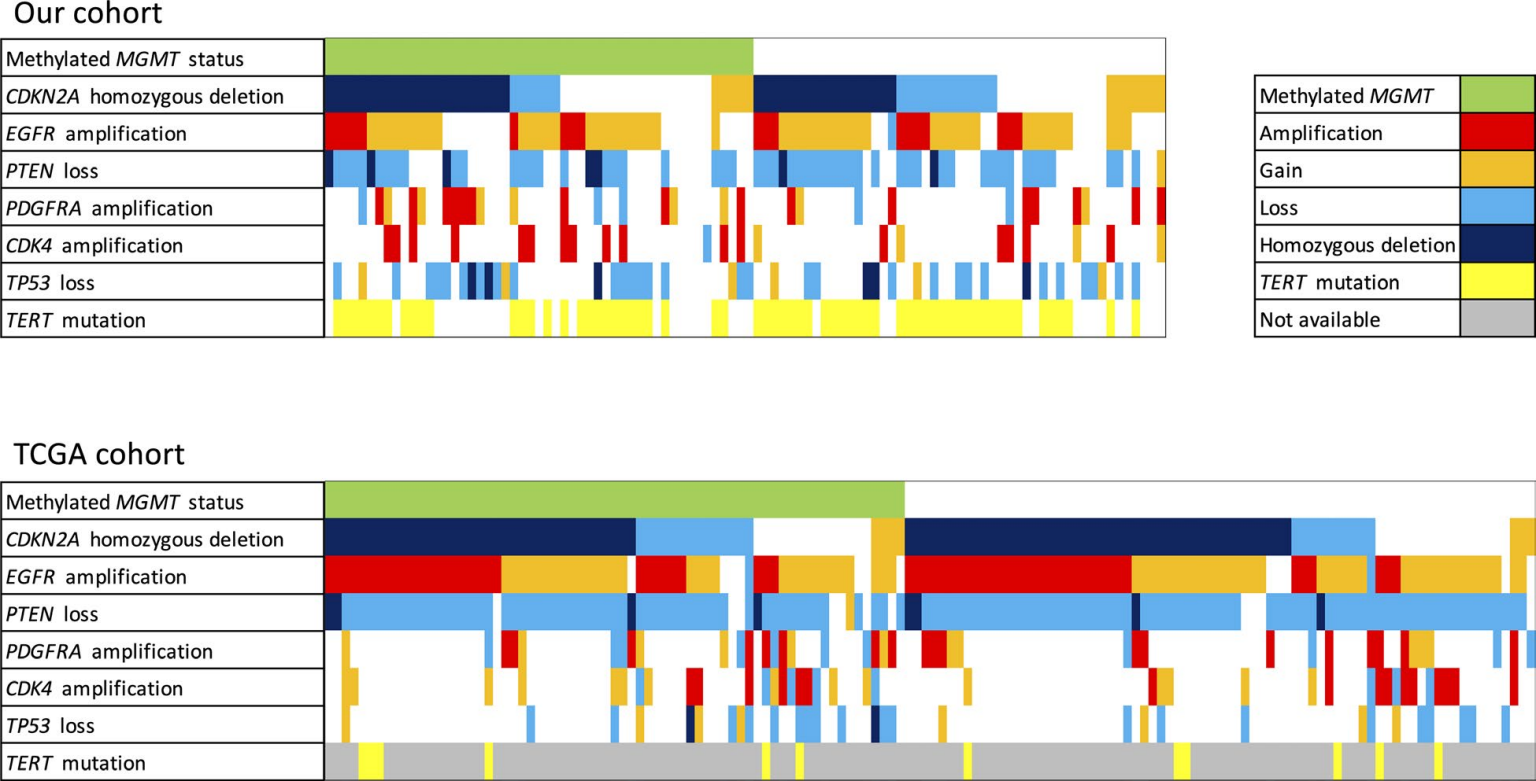
Comparison of genetic distribution in *IDH*‐wildtype GBM between the two cohorts. The diagram shows the landscape of the molecular characteristics of *IDH*‐wildtype GBM from our cohort and TCGA cohort

**FIGURE 3:**
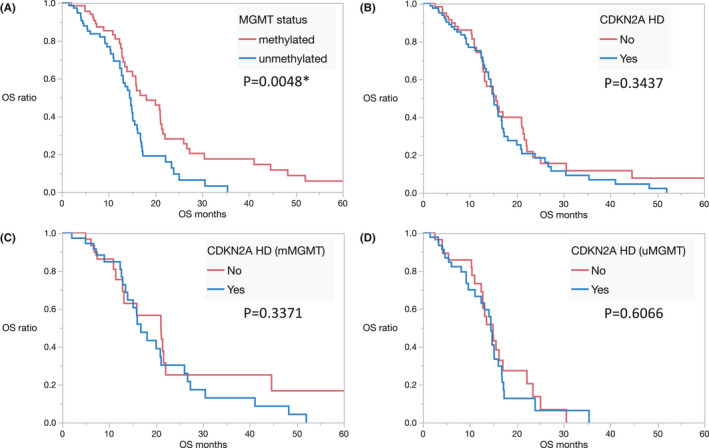
(A) Kaplan–Meier OS estimates in patients of newly diagnosed GBM in the TCGA cohort. Patients with methylated and unmethylated *MGMT* status are represented by the red and blue lines, respectively. (B) Kaplan–Meier OS estimates in patients of newly diagnosed GBM in the TCGA cohort. Patients without and with *CDKN2A* homozygous deletion are represented by the red and blue lines, respectively. (C) Kaplan–Meier OS estimates in patients of newly diagnosed GBM with methylated *MGMT* status in the TCGA cohort. Patients without and with *CDKN2A* homozygous deletion are represented by the red and blue lines, respectively. (D) Kaplan–Meier OS estimates in patients of newly diagnosed GBM with unmethylated *MGMT* status in the TCGA cohort. Patients without and with *CDKN2A* homozygous deletion are represented by the red and blue lines, respectively

As a validation study, we analyzed the data of 144 patients with *IDH*‐wildtype GBM in TCGA. Figure 2 shows a comparison of the genetic distributions in *IDH*‐wildtype GBM between our cohort and the TCGA cohort. In the TCGA cohort, *TERT* mutation data were not available. The frequency of *EGFR* amplification and *CDKN2A* homozygous deletion in the TCGA cohort was higher than in this study. Furthermore, in the TCGA cohort, the median OS of patients with methylated and unmethylated *MGMT* status was significantly different (18.1 and 14.5 months, respectively; *p* = 0.0048) (Figure 3A), but not the median OS for patients with and without *CDKN2A* homozygous deletion (15.1 and 15.6 months, respectively; *p* = 0.3437) (Figure 3B). In patients with methylated *MGMT* status, there was no significant difference between the median OS of patients with and without *CDKN2A* homozygous deletion (16.8 and 21.1 months, respectively; *p* = 0.3529) (Figure 3C), nor was there a difference between the median OS of patients with and without *CDKN2A* homozygous deletion in patients with unmethylated *MGMT* status (14.5 and 14.9 months, respectively; *p* = 0.6066) (Figure 3D). Consequently, the three prognostic groups in our cohort could not be validated in the TCGA cohort.

### 3.4 Comparison between pre‐BEV and post‐BEV eras

**FIGURE 4:**
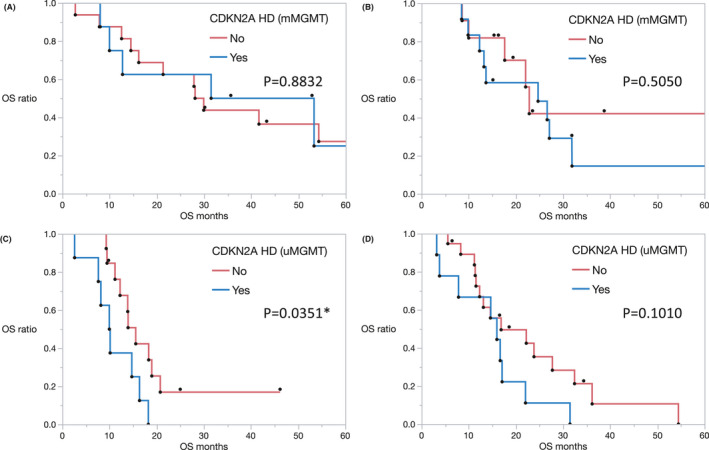
(A) Kaplan–Meier OS estimates in patients of newly diagnosed GBM with methylated *MGMT* status in our cohort in the pre‐BEV era. Patients without and with *CDKN2A* homozygous deletion are represented by the red and blue lines, respectively. (B) Kaplan–Meier OS estimates in patients of newly diagnosed GBM with methylated *MGMT* status in our cohort in the post‐BEV era. Patients without and with *CDKN2A* homozygous deletion are represented by the red and blue lines, respectively. (C) Kaplan–Meier OS estimates in patients of newly diagnosed GBM with unmethylated *MGMT* status in our cohort in the pre‐BEV era. Patients without and with *CDKN2A* homozygous deletion are represented by the red and blue lines, respectively. (D) Kaplan–Meier OS estimates in patients of newly diagnosed GBM with unmethylated *MGMT* status in our cohort in the post‐BEV era. Patients without and with *CDKN2A* homozygous deletion are represented by the red and blue lines, respectively. CDKN2A homozygous deletion showed no significant impact on OS in patients with methylated MGMT status, while, among patients with unmethylated MGMT status, there was a significant difference in OS between patients with and without CDKN2A homozygous deletion. This difference was more evident in the pre‐BEV era, but has become non‐significant in the post‐BEV era due to OS improvement in patients with CDKN2A homozygous deletion

Although significant bias was identified in terms of preoperative KPS score (*p* = 0.0036) and maximum tumor diameter (*p* = 0.0111) between the two eras, no other significant differences were observed (Table 1). In patients with methylated *MGMT* status in the pre‐BEV and post‐BEV eras, no significant difference was observed between patients with and without *CDKN2A* homozygous deletion (Figure 4A, *p* = 0.8832; Figure 4B, *p* = 0.5050). On the other hand, in patients with unmethylated *MGMT* status in the pre‐BEV era, there was a significant difference in the median OS of patients with and without *CDKN2A* homozygous deletion (10.1 and 15.6 months, respectively; *p* = 0.0351) (Figure 4C). However, this difference became non‐significant in the post‐BEV era (median OS: 16.0 and 16.9 months, respectively; *p* = 0.1010) (Figure 4D) due to the OS improvements in patients with *CDKN2A* homozygous deletion.

## 4 DISCUSSION

In this study, we investigated the impact of *CDKN2A* homozygous deletion as a prognostic marker in combination with methylated *MGMT* status for patients with *IDH*‐wildtype GBM. Our results indicated that molecular classification based on methylated *MGMT* status and *CDKN2A* homozygous deletion defines three prognostic groups. Methylated *MGMT* status is recognized as the most robust predictive marker in patients with GBM; however, there is insufficient evidence regarding the impact of *CDKN2A* homozygous deletion on OS for *IDH*‐wildtype GBM, and therefore, its prognostic impact remains controversial.^20^ Umehara et al. reported that genetic markers such as *EGFR*, *CDKN2A*, and *PTEN* commonly show a prognostic value when combined, although the CNAs for these genetic markers did not significantly affect patients’ clinical outcomes by themselves.^37^ In this study, after accounting for the effect of methylated *MGMT* status, we investigated the impact of *CDKN2A* homozygous deletion on patients with *IDH*‐wildtype GBM. While methylated *MGMT* status was associated with higher sensitivity with respect to TMZ therapy, leading to a favorable prognosis, *CDKN2A* homozygous deletion seemed to result in aggressive bioactivity affecting OS impact in patients with unmethylated *MGMT* status in our cohort. Future development of novel chemotherapeutic agents targeting *CDKN2A* alternation would be a promising approach not only for *IDH*‐mutant gliomas but also for GBMs with unmethylated *MGMT*.

In this study, although the OS of patients with unmethylated *MGMT* was significantly different between patients with and without *CDKN2A* homozygous deletion in the pre‐BEV era, this difference became less relevant in the post‐BEV era due to the OS improvement in patients with *CDKN2A* homozygous deletion. BEV, an inhibitor of vascular endothelial growth factor (VEGF), was approved for the treatment of multiple cancers by the Food and Drug Administration (FDA), and after two phase 2 clinical trials, in 2009, the FDA approved BEV for the treatment of recurrent GBM.^38^, ^39^ Thereafter, the AVAglio and RTOG 0825 phase 3 randomized clinical trials proved that BEV improved the progression‐free survival (PFS) of patients with newly diagnosed GBM.^27^, ^40^ Although OS prolongation was not confirmed in this clinical trial, and the clinical benefit of BEV for GBM remains controversial, this trial led to BEV being approved in Japan as an insurance‐covered first‐line drug for GBM, in 2013, concurrently with its second‐line application. In our institution, since its approval, BEV has been used in combination with the Stupp regimen for patients with severe clinical conditions such as unresectable tumors or low KPS scores; the remaining patients are treated in accordance with the Stupp regimen and with second‐line BEV after recurrence. We previously reported the clinical benefit of such optional first‐line administration of BEV, complementary to TMZ therapy,^22^ and highlighted the advantages of first‐line BEV treatment for severe clinical conditions, such as unresectable tumors. The prolongation of survival time in patients with unresectable tumors is based on the hypothesis that BEV contributes to an improved residual tumor control for progressive disease, which is important in the context of improving real‐world outcomes. Since BEV approval for GBM treatment, several predictive BEV markers have been reported; but with an insufficient level of evidence. To validate the usefulness of these markers, further real‐world data need to be accumulated and validated. *EGFR* amplification and classical subtype were reported to be associated with poor response to BEV.^41^ In addition, we previously reported that the therapeutic sensitivity of BEV is high in patients with unmethylated *MGMT* status with poor prognosis.^22^ AVAglio sub‐analysis similarly suggested that BEV’s impact on OS manifested only in patients with newly diagnosed GBM with proneural *IDH*‐wildtype tumors, which was associated with poorer prognosis in the cohort.^42^ In this study, although a significant difference in OS was observed between the intermediate prognosis and poor prognosis groups in the pre‐BEV era, there was no difference between these two groups in the post‐BEV era. This outcome indicates the impact of BEV approval on patients with unmethylated *MGMT* status, particularly on patients in the poor prognosis group harboring *CDKN2A* homozygous deletion.

This study has several limitations. First, it was a non‐randomized, retrospective observational study, and similar outcomes as those in our cohort were not obtained in the TCGA validation cohort. This discrepancy may be due to selection bias between the two cohorts. Although we selected patients with backgrounds similar to those of patients in the TCGA cohort, there were differences in molecular profile frequencies (Figure 2). One possible reason for this bias is race differences, since lower *EGFR* amplification rates in patients with GBM from Asia were recently reported during a screening for the INTELLANCE1 and INTELLANCE2 randomized GBM trials for depatux‐m.^43^ As the frequencies of molecular profiles in our cohort were similar to those in the Kansai Molecular Diagnosis Network for CNS tumors,^37^ which is another Japanese cohort, it is possible that Japanese patients with GBM may have unique genetic characteristics. Another possible reason is that the TCGA cohort included both targeted and genome‐wide screens. Because targeted screens in the TCGA cohort only analyzed specific genetic markers, more comprehensive validation is necessary in future studies.

Second, differences in molecular biology techniques should also be considered. We confirmed CNAs using MLPA, which has a lower output than the comprehensive high‐throughput array used for the TCGA cohort.^37^ In addition, the limited genetic analysis in our cohort could have overlooked other distinct genetic markers which might have influenced the study's outcomes. Current technologies have revealed huge genetic profiling whose clinical significance is mostly still uncertain. Hamid et al. reported the function and tissue distribution of genes flanking the *CDKN2A* locus, and demonstrated that one of these genes, *MTAP*, which is essential for adenosine monophosphate and methionine salvage, frequently co‐deleted with *CDKN2A* homozygous deletion.^44^ Satomi et al. reported the loss of MTAP immunohistochemical staining due to *MTAP* homozygous deletion as a surrogate marker of *CDKN2A* homozygous deletion, although there were several concerns regarding the interpretation and performance of MTAP immunohistochemistry.^45^ In the TCGA cohort of this study, 66 of 67 (98.5%) patients with *MTAP* homozygous deletion harbored *CDKN2A* homozygous deletion; however, 66 of 83 (79.5%) patients with *CDKN2A* homozygous deletion harbored *MTAP* homozygous deletion (data not shown). Although the clinical impact of this discrepancy between *CDKN2A* and *MTAP* status is currently unclear, such regulator genes in the vicinity of *CDKN2A* may have impacted the outcomes in our cohort.

Third, the other limitation was the inconsistency in treatment regimens. Treatment approaches for GBM have changed over time, and this may have influenced treatment outcomes. Developments related to awake surgery, intraoperative support devices, navigation (e.g., fluorescence‐guided surgery with 5‐aminolevulinic acid), chemotherapy, and radiotherapy could also have improved the treatment outcomes of GBM. While the FDA has approved BEV use only for recurrent GBM, the use of first‐line BEV concurrently with its second‐line application is approved only in Japan. As our institution has adapted first‐line BEV use for patients with severe clinical conditions since BEV approval, such optimization of BEV application might have impacted the outcomes in our cohort as well. Further accumulation of clinical data and evidence, such as ours, is warranted to evaluate the real‐world impact of BEV.

## 5 CONCLUSIONS

We classified outcomes of *IDH*‐wildtype GBM outcomes into three groups, as follows—good prognosis: for patients with methylated *MGMT* status, intermediate prognosis: for patients with unmethylated *MGMT* status and without *CDKN2A* homozygous deletion, and poor prognosis: for patients with unmethylated *MGMT* status and *CDKN2A* homozygous deletion. In patients in the good prognosis group, with methylated *MGMT* status, a higher therapeutic effect of TMZ can be expected. Although the prognosis is poor in the group with unmethylated *MGMT* status and *CDKN2A* homozygous deletion, BEV administration seems to be, at least, partly beneficial.

This study was approved by a local ethics committee (Kyushu University Institutional Review Board for Clinical Research: 2019–90) and conducted in accordance with the 1964 Declaration of Helsinki (as revised in Fortaleza, Brazil, October 2013).

## CONFLICT OF INTEREST

The authors report no conflict of interest concerning the materials or methods used in this study or the findings specified in this paper.

## Data Availability

The data that support the findings of this study are available on request from the corresponding author. The data are not publicly available due to privacy or ethical restrictions.
